# Emergency Department Utilization by Children in the USA, 2010–2011

**DOI:** 10.5811/westjem.2017.7.33723

**Published:** 2017-09-26

**Authors:** Tadahiro Goto, Kohei Hasegawa, Mohammad Kamal Faridi, Ashley F. Sullivan, Carlos A. Camargo

**Affiliations:** Harvard Medical School, Massachusetts General Hospital, Department of Emergency Medicine, Boston, Massachusetts

## Abstract

**Introduction:**

Epidemiological surveillance data for emergency department (ED) visits by children are imperative to guide resource allocation and to develop health policies that advance pediatric emergency care. However, there are sparse population-based data on patient-level information (e.g., the number of children who present to the emergency department [ED]). In this context, we aimed to investigate both the patient- and visit-level rates of ED utilization by children.

**Methods:**

This was a retrospective cohort study using population-based multipayer data – state ED databases (SEDD) and state inpatient databases (SID) – from six geographically-dispersed U.S. states (California, Florida, Iowa, Nebraska, New York, and Utah) in 2010 and 2011. We identified all children aged <18 years who presented to the ED and described the patient-level ED visit rate, visit-level ED visit rate, and proportion of all ED visits made by children. We conducted the analysis using the 2011 SEDD and SID data. We also repeated the analysis using the 2010 data to determine the consistency of the results across different years.

**Results:**

In 2011, 2.9 million children with a patient identifier presented to EDs in the six U.S. states. At the patient-level, 15 out of every 100 children presented to an ED at least once per year. Of these children, 25% presented to EDs 2–3 times per year with an approximately 1.5-fold variation across the states (e.g., 19% in Utah vs. 28% in Florida). In addition, 5% presented to EDs ≥4 times per year. At the visit-level, 6.7 million ED visits were made by children in 2011 – 34 ED visits per 100 children annually. ED visits by children accounted for 22% of all ED visits (including both adults and children), with a relatively small variation across the states (e.g., 20% in New York vs. 24% in Nebraska). Analysis of the 2010 data gave similar results for the ED utilization by children.

**Conclusion:**

By using large population-based data, we found a substantial burden of ED visits at both patient- and visit-levels. These findings provide a strong foundation for policy makers and professional organizations to strengthen emergency care for children.

## INTRODUCTION

Approximately 30 million emergency department (ED) visits are made by children in the U.S. annually.^1^,^2^ ED visits by children increased 14.4% between 2001 and 2010. 3 The increased burden of ED visits by children underscores the importance of pediatric emergency care readiness. 4 Although substantial efforts have improved the pediatric readiness over years, the state of emergency care readiness for U.S. children remains insufficient and uneven due to resource and workforce disparities. 5 For example, recent U.S. surveillance data reported that only 45% of EDs have a quality improvement plan addressing the needs of children 5 and that 17%–65% of EDs have a physician or nurse pediatric emergency care coordinator — who ensures that staffs are appropriately trained, that the ED is prepared with the appropriate equipment, and that the right kinds of policies are in place for caring for children.^4^,^6^

To guide appropriate resource allocation and advance pediatric emergency care, surveillance data of the current ED visits made by children are instrumental. A combination of the patient- and visit-level information on the ED visits provides a comprehensive view of ED utilization by children. Although prior reports have documented visit-level information for U.S. children (e.g., the number of ED visits and visit-level ED visit rate),^1^,^2^ there are limited data on patient-level data (e.g., the number of children who present to the ED). 7–9 An analysis of the National Health Interview Survey reported that 12% of children living in the U.S. (8.8 million children) had an ED visit in the preceding 12 months in 2012; approximately half of these children (4.2 million children) had two or more ED visits. 7 However, this study is potentially limited by the low response rate (70%), lack of onsite verification, and recall bias. 7 Two other multicenter pediatric ED studies have reported that 36%–38% of children who visited the ED had multiple ED visits.^8^,^9^ Yet, since 90% of children are brought to general EDs, 10 focusing on high-volume pediatric centers might cause selection bias.

To address the knowledge gap, we aimed to investigate both the patient- and visit-level rates of ED utilization by children in six geographically-dispersed U.S. states by using population-based multipayer datasets.

## METHODS

We conducted a retrospective cohort study using 2010–2011 data from the Healthcare Cost and Utilization Project (HCUP) state emergency department databases (SEDD) and state inpatient databases (SID) from six U.S. states (California, Florida, Iowa, Nebraska, New York, and Utah). Details of the methods may be found in the supplemental material. The SEDD includes all treat-and-release and transfer ED visits from short-term, acute-care, nonfederal, community hospitals in participating states. The SID includes all inpatient discharges from short-term, acute-care, nonfederal, general, and other specialty hospitals in participating states, including those hospitalized from the ED. Taken together, we identified all ED visits regardless of disposition. Further information on SEDD and SID databases can be found elsewhere. 11 In the current study, these six states were selected for data availability, their geographic distribution, high data quality, and chiefly because their databases contain unique patient identifiers that enable follow-up of individual patients across years.

We identified all children aged <18 years who presented to the ED in the six states during 2010–2011. We investigated 1) the patient-level ED visit rate (i.e., the number of children who presented to EDs per 100 children), 2) the visit-level ED visit rate (i.e., the number of ED visits by children per 100 children), and 3) the proportion of ED visits made by children among all ED visits (including both children and adults). The denominators for ED visit rates were the population estimates obtained from the U.S. Census Bureau. 12 For the patient-level analysis, we excluded children with no record of a patient identifier. Descriptive statistics were performed using Stata version14.1 (StataCorp, College Station, TX). First, we conducted the analysis using data from the 2011 SEDD and SID. Next, we repeated this analysis using data from the 2010 data to determine the consistency of the results across the different years. The institutional review board of Massachusetts General Hospital approved this analysis.

## RESULTS

In 2011, 2.9 million children with a patient identifier presented to EDs in the six U.S. states (Table 1). Children in California, Florida, and New York accounted for approximately 90% of these children. Overall, the median age was eight years, 39% were non-Hispanic White, and 52% were Medicaid beneficiaries at the first ED visit. Compared to children who presented to the ED once per year, those who presented to the ED multiple times per year were younger, and more likely to be female sex, non-Hispanic Black or Hispanic, Medicaid beneficiaries, and living in the area with lower median household income (all P<0.001; Table 1). At the patient-level, 15 out of every 100 children presented to an ED at least once per year. Of these children, 25% presented to EDs 2–3 times per year with an approximately 1.5-fold variation across states (e.g., 19 % in Utah vs. 28% in Florida). Additionally, 5% had ≥4 ED visits per year.

At the visit-level, 6.7 million ED visits were made by children in 2011 – 34 ED visits per 100 children annually (Table 2). There was an approximate two-fold variation across states (e.g., 19 visits in Utah vs. 43 visits in Florida, per 100 children annually). ED visits by children accounted for 22% of all ED visits (including both children and adults), with a relatively small variation across states (e.g., 20% in New York vs. 24% in Nebraska).

The analysis of the 2010 data gave similar results for the burden of ED visits by children (Supplemental Table 1). At the patient-level, 14 out of every 100 children presented to an ED at least once per year; of these, 24% presented to EDs 2–3 times per year, and 5% had ≥4 ED visits per year. At the visit-level, there were 33 ED visits per 100 children annually, an d ED visits by children accounted for 22% of all ED visits.

## DISCUSSION

In this analysis of population-based multipayer data from the six U.S. states, at the patient-level one in seven children presented to the ED at least once per year. Additionally, 25% of these children had 2–3 ED visits and 5% had ≥4 ED visits. At the visit-level, children accounted for 22% of all ED visits, consistent with the estimates using nationally-representative databases — e.g., the Nationwide Emergency Department Sample ED visits by children accounted for 20% of all ED visits in 2011. 13 Our population-based data corroborates the previous reports on the visit-level data, but our study then extends beyond them by investigating ED utilization at the patient-level.

In the U.S., EDs serve as a primary safety net, an acute diagnostic and treatment center, and a 24/7 portal for rapid hospitalization. The visit-level findings indicate that the burden of ED visits by children continued to be substantial, accounting for approximately 20% of all ED visits from 1997 to 2011.^1^,^2^,^14^ Consistent with our findings, the previous patient-level analysis revealed that 12%–14% of U.S. children visited the ED at least once per year. 7 In contrast, the proportion of children with ≥2 ED visits within a year in this population-based study (i.e., 30%) was lower compared to studies using interview or multicenter registries – approximately 40% of children who visited the ED had repeat ED visits. 7,8,9 This discrepancy might be attributable to the difference in study design, population, data measurement, or any combination of these factors. Nevertheless, the validity of the current findings is supported by the use of population-based databases that captured all ED visits and patients across six U.S. states.

We also found the differences in demographics and socioeconomic status between children who presented to the ED once per year and those who presented multiple times per year. For example, children with multiple ED visits were more likely to be racial/ethnic minorities, Medicaid beneficiaries, and living in the area with lower median household income. Although further investigation is warranted, these findings suggest the potential relationship between socio-demographics and frequent ED utilization – e.g., lower socioeconomic status might be associated with the frequent ED utilization among children.

The observed large ED utilization by children necessitates the appropriate allocation of resources and improvement of guidelines-recommended pediatric readiness. The national guidelines of pediatric readiness target seven areas of focus^15^: 1) administration and coordination, 2) physicians, nurses, and other healthcare clinicians, 3) quality improvement, 4) patient safety, 5) policies, procedures, and protocols, 6) support services, and 7) equipment, supplies, and medications.^16^ The first area of these focuses – the availability of pediatric emergency care coordinators – has been reported to be insufficient.^5^ While the Institute of Medicine has recommended that all hospitals – regardless of the pediatric ED visit volume – should have pediatric emergency care coordinators,^4^ studies have reported that 17%–65% of EDs do not have a physician or nurse pediatric emergency care coordinator.^4^,^6^ As for physicians’ training, particularly for emergency medicine residency training, the Accreditation Council for Graduate Medical Education set the requirement that 20% of all ED encounters should be dedicated to the care of pediatric patients.^17^ This requirement is consistent with our observation that 20%–22% of overall ED visits are made by children.^13^ By contrast, while mandatory pediatric emergency care competency evaluations are recommended in the aforementioned guidelines,^15^ the competency evaluations are not often conducted either in midlevel staff (18.1%) or in physicians (38.7%).^5^ In terms of the quality improvement in pediatric care, a previous survey demonstrated that only 45% of EDs reported having a quality improvement plan addressing the needs of children.^5^ Additionally, almost 50% of EDs had barriers to guideline implementations due to the cost of training and the lack of educational resources.^5^ Because of children’s unique healthcare needs, our findings in conjunction with the literature^5^ suggest that these critical chasms in the pediatric readiness in the ED should be the priority area for improvement efforts.^4^

## LIMITATIONS

This study has several potential limitations. First, this study population was not a random sample of the entire U.S. The overall findings are largely attributable to the data from three large-population states (California, Florida, and New York) and there was a lack of information from some U.S. regions, such as the Pacific Northwest. Second, the lack of a patient identifier in some children may have led to an underestimation of the patient-level ED visit rate – that is, the current study indicates the “least” patient-level ED visit rates. Third, although the age cut-off to define pediatric population varies across studies,^18^–^20^ we defined children as age ≤18 years to maintain consistency with the methods used in the national estimates of ED visits made by children.^13^,^21^ Finally, this study focused on ED utilization in acute care hospitals; we recognize that many children who need emergency care might have presented to other settings (e.g., urgent care centers). Thus, observed findings do not represent the total burden of children who need unique care. Nevertheless, as we focused on the patient- and visit-level ED visits, our observations are highly relevant to millions of U.S. children visiting the ED and their families.

## CONCLUSION

In summary, this study of the large population-based multipayer databases from six U.S. states found that one in seven children presented to the ED at least once per year, and that 30% of these children had multiple ED visits within a single year. This patient-level information provides detailed information that characterizes the children who present to the ED. The observed data also indicated that children accounted for 22% of all ED visits in these states. This visit-level information indicates the actual burden of ED use by children. In addition, the patient-level and visit-level ED visit rates varied widely across the study states. Given the large ED utilization by children, our data should encourage healthcare providers, hospitals, professional organizations, policymakers, and other stakeholders involving emergency care for children to continue their efforts for improving pediatric readiness in the ED.

## Supplementary Information





## Figures and Tables

**Table 1:** Baseline characteristics of children who presented to an emergency department, at first visit in 2011.

Characteristics	Children who presented to ED once per year^1^	Children who presented to ED 2–3 times per year^1^	Children who presented to ED ≥4 times per year^1^	p value*
Age (year), median (IQR)	9 (3–15)	7 (2–14)	5 (1–14)	<0.001
Male sex	1,054,016 (53%)	361,728 (51%)	69,205 (49%)	<0.001
Race/ethnicity				<0.001
Non-Hispanic white	762,134 (41%)	237,888 (36%)	46,064 (34%)	
Non-Hispanic black	376,225 (20%)	152,602 (23%)	33,461 (24%)	
Hispanics	555,099 (30%)	220,202 (33%)	46,945 (34%)	
Others	184,853 (10%)	58,407 (9%)	10,803 (8%)	
Primary health insurance				<0.001
Medicare	6,734 (1%)	2,364 (1%)	507 (1%)	
Medicaid	945,377 (50%)	428,473 (61%)	102,074 (71%)	
Private	791,610 (39%)	187,634 (27%)	24,696 (17%)	
Self-pay	191,148 (9%)	60,289 (9%)	10,969 (8%)	
Others	79,428 (4%)	26,383 (4%)	4,833 (3%)	
Quartiles for median household income				<0.001
1 (lowest)	676,327 (34%)	274,490 (39%)	61,807 (44%)	
2	530,429 (27%)	193,803 (28%)	39,844 (28%)	
3	443,820 (22%)	143,303 (21%)	26,652 (19%)	
4 (highest)	336,064 (17%)	84,309 (12%)	12,703 (9%)	
Patient residence				<0.001
Metropolitan area	1,824,303 (91%)	636,926 (90%)	128,921 (90%)	
Rural area	186,595 (9%)	67,711 (10%)	14,106 (10%)	

*ED*, emergency department; *IQR*, interquartile range.

1 Children with a patient identifier.

* Comparison between children who presented to ED once per year and those who presented to ED ≥2 times per year.

**Table 2:** Annual child-related emergency department visits and rates in six U.S. states in 2011.

States (year 2011)	Patient-level				Visit-level		
	
	Total number of children who presented to ED, n^1^	Total number of children who presented to ED 2–3 times per year, n (%)	Total number of children who presented to ED ≥4 times per year, n (%)	Number of children who presented to ED per 100 person-years^2^	Total number of pediatric ED visits, n	Pediatric ED visit rate per 100 person-years^2^	Proportion of pediatric ED visits among all ED visits^3^
Overall	2,863,705	705,445 (25)	143,220 (5)	15	6,696,967	34	22%
California	847,646	205,088 (24)	39,412 (5)	9	2,752,545	30	23%
Florida	734,933	204,929 (28)	47,919 (7)	18	1,730,086	43	20%
Iowa	67,898	16,469 (24)	3,415 (5)	9	259,074	36	22%
Nebraska	97,781	21,089 (22)	3,580 (4)	22	130,396	28	24%
New York	1,071,372	249,535 (23)	47,364 (4)	25	1,654,211	39	20%
Utah	44,075	8,335 (19)	1,455 (3)	5	170,655	19	23%

*ED*, emergency department.

1 Children with a patient identifier (39% of ED visits had no patient identifier).

2 Denominators were pediatric population (age <18 years) in each state.

3 Denominators were all adults and pediatric population in each state.
