# Supplementary material for: Emergency Department Utilization by Children in the USA, 2010–2011
Source: West J Emerg Med. 2017 Sep 26;18(6):1042–6. doi: 10.5811/westjem.2017.7.33723 (PMC5654872; doi:10.5811/westjem.2017.7.33723)
Supplement: Supplementary file 1 [file wjem-18-1042-s001.docx]

**Supplemental Table 1.** Annual child-related emergency department visits and rates in six U.S. states, 2010

|  | Patient-level | | | | Visit-level | | |
| --- | --- | --- | --- | --- | --- | --- | --- |
| States | Total number of children who presented to ED, n^1^ | Total number of children who presented to ED 2-3 times per year, n (%) | Total number of children who presented to ED ≥4 times per year, n (%) | Number of children who presented to ED per 100 person-years^2^ | Total number of pediatric ED visits, n | Pediatric ED visit rate per 100 person-years^2^ | Proportion of pediatric ED visits among all ED visits^3^ |
| **2010** |  |  |  |  |  |  |  |
| Overall | 2,853,739 | 696,563 (24) | 140,917 (5) | 14 | 6,550,836 | 33 | 22% |
| California | 854,465 | 206,064 (24) | 39,926 (5) | 9 | 2,711,794 | 29 | 23% |
| Florida | 734,831 | 203,928 (28) | 47,555 (6) | 18 | 1,677,904 | 42 | 21% |
| Iowa | 65,033 | 15,224 (23) | 3,130 (5) | 9 | 247,704 | 34 | 22% |
| Nebraska | 98,684 | 21,063 (21) | 3,561 (4) | 22 | 130,658 | 28 | 24% |
| New York | 1,056,731 | 241,517 (23) | 45,371 (4) | 24 | 1,614,347 | 37 | 20% |
| Utah | 43,995 | 8,767 (20) | 1,374 (3) | 4 | 168,429 | 19 | 23% |

^1^Children with a patient identifier (39% of ED visits had no patient identifier)

^2^Denominators were pediatric population (age <18 years) in each state

^3^Denominators were all adults and pediatric population in each state

*ED*, emergency department
