# Supplementary material for: Emergency Department Utilization by Children in the USA, 2010–2011
Source: West J Emerg Med. 2017 Sep 26;18(6):1042–6. doi: 10.5811/westjem.2017.7.33723 (PMC5654872; doi:10.5811/westjem.2017.7.33723)
Supplement: Supplementary file 2 [file wjem-18-1042-s002.docx]

**Supplemental Material (eMethods)**

**Emergency Department Utilization by Children in the US, 2010-2011**

**Tadahiro Goto, MD, MPH**

**Kohei Hasegawa, MD, MPH**

**Mohammad Kamal Faridi, MPH**

**Ashley F. Sullivan, MPH, MS**

**Carlos A. Camargo, Jr., MD, DrPH**

Harvard Medical School, Massachusetts General Hospital, Department of Emergency Medicine, Boston, Massachusetts

**Methods**

We conducted a retrospective cohort study using 2010 and 2011 data from the Healthcare Cost and Utilization Project (HCUP) State Emergency Department Databases (SEDD) and State Inpatient Databases (SID) from six US states (California, Florida, Iowa, Nebraska, New York, and Utah). The HCUP is a family of health care databases developed through a federal-state-industry partnership and sponsored by the Agency for Healthcare Research and Quality. The HCUP databases bring together the data collection efforts of State data organizations, hospital associations, private data organizations, and the Federal government to create a national information resource of encounter-level health care data. These HCUP databases enable research on a broad range of health policy issues, including medical practice patterns, access to health care programs, and outcomes of treatments at the national, state, and local levels. The SEDD includes all treat-and-release and transfer ED visits from short- term, acute-care, nonfederal, community hospitals in participating states. The SEDD does not include urgent care data. The ED encounter abstracts from hospital-affiliated EDs in the participating States are translated into a uniform format to facilitate multistate comparisons and analyses. The SID contains all inpatient discharges from short-term, acute-care, nonfederal, general, and other specialty hospitals in participating states, including those hospitalized from the ED. The SID encompass more than 95 percent of all US hospital discharges. The abstracts in the SID are also translated into a uniform format. The SEDD and SID contain clinical and administrative variables included in a hospital discharge abstract, such as the principal and secondary diagnoses and procedures, admission and discharge status, patient demographics characteristics (e.g., sex, age, and, for some States, race/ethnicity), and payer. Among patients who presented to EDs, non-hospitalized patients are included in SEDD and admitted patients are included in SID database (i.e., ED data of the SEDD and SID are mutually exclusive). Taken together, we identified all ED visits, regardless of disposition, in the study states. Further information on the SEDD and SID databases can be found elsewhere.[^1^](#_ENREF_1) In the current study, these six states were selected for their geographic distribution, high-quality data, and chiefly because their databases contain unique encrypted patient identifiers that enable follow-up of specific patients across years.

All children aged <18 years who presented to the ED in the six states during 2010-2011 were included in the study. In cases where age was missing, patient’s age was identified by using another ED visit record of the same patient that had information on age. We investigated 1) the patient-level ED visit rate (i.e., the number of children who presented to EDs per 100 children), 2) the visit-level ED visit rate (i.e., the number of ED visits by children per 100 children), and 3) the proportion of ED visits made by children among all ED visits (including both children and adults). The denominators for ED visit rates were the population estimates for each state in each of the studied year (2010 and 2011) obtained from the US Census Bureau.[^2^](#_ENREF_2) For the patient-level analysis, we excluded children with no patient identifier. Descriptive statistics were performed using Stata version 14.1 (StataCorp, College Station, TX). First, we conducted the analysis using data from the 2011 SEDD and SID. Next, we repeated this analysis using the 2010 data to assess the consistency of the results across the different years because a single-year study may not have sufficient validity on the frequency of ED visits (e.g., flu pandemic in a specific year). The institutional review board of Massachusetts General Hospital approved this analysis.

**REFERENCES**

1. Overview of the State Inpatient Databases (SID). Healthcare Cost and Utilization Project. Agency for Healthcare Research and Quality. Available at: <http://www.hcup-us.ahrq.gov/sidoverview.jsp>. Accessed September 15, 2016.

2. US Census Bereau. Population estimates. Available at: <http://www.census.gov/popest/>. Accessed September 15, 2016.
